# Cyanobacterial Bloom in Urban Rivers: Resource Use Efficiency Perspectives for Water Ecological Management

**DOI:** 10.3390/microorganisms13091981

**Published:** 2025-08-25

**Authors:** Qingyu Chai, Yongxin Zhang, Yuxi Zhao, Hongxian Yu

**Affiliations:** 1 Department of Management, Heilongjiang University of Science and Technology, Harbin 150022, China; chaiqingyu@outlook.com (Q.C.);; 2 College of Wildlife and Protected Area, Northeast Forestry University, Harbin 150040, China

**Keywords:** urban river, cyanobacterial blooms, resource use efficiency, nutrient thresholds, ecological management

## Abstract

Cyanobacterial blooms in urban rivers present critical ecological threats worldwide, yet their mechanisms in fluvial systems remain inadequately explored compared to lacustrine environments. This study addresses this gap by investigating bloom dynamics in the eutrophic Majiagou River (Harbin, China) through phytoplankton resource use efficiency (RUE), calculated as chlorophyll-a per unit TN/TP. Seasonal sampling (2022–2024) across 25 rural-to-urban sites revealed distinct spatiotemporal patterns: urban sections exhibited 1.9× higher cyanobacterial relative abundance (RAC, peaking at 40.65% in autumn) but 28–30% lower RUE than rural areas. Generalized additive models identified nonlinear RAC–RUE relationships with critical thresholds: in rural sections, RAC peaked at TN-RUE 40–45 and TP-RUE 25–30, whereas urban sections showed lower TN-RUE triggers (20–25) and suppressed dominance above TP-RUE 10. Seasonal extremes drove RUE maxima in summer and minima during freezing/thawing periods. These findings demonstrate that hydrological stagnation (e.g., river mouths) and pulsed nutrient inputs reduce nutrient conversion efficiency while lowering bloom-triggering thresholds under urban eutrophication. The study establishes RUE as a predictive indicator for bloom risk, advocating optimized N/P ratios coupled with flow restoration rather than mere nutrient reduction. This approach provides a science-based framework for sustainable management of urban river ecosystems facing climate and anthropogenic pressures.

## 1. Introduction

The frequent occurrence of cyanobacterial blooms has become a severe challenge for global freshwater ecosystems, especially in eutrophic lakes [1]. Excessive nutrient inputs caused by human activities such as agricultural production and wastewater discharge lead to the overproliferation of harmful algae like cyanobacteria, posing serious threats to water quality and ecological balance. The occurrence of cyanobacterial blooms results in decreased dissolved oxygen levels in water bodies and the release of harmful substances such as algal toxins (e.g., microcystins), threatening the ecosystem services of urban rivers and human health [2]. The formation mechanisms of cyanobacterial blooms and their impacts on ecosystem structure and function are complex and variable, often exhibiting seasonal changes [3,4]. Bloom formation is influenced not only by nutrient accumulation but also by climate change and hydrological conditions. The hydrological characteristics of the Majiagou River indicate low flow velocity, especially during the high-temperature summer period, further accelerating algal reproduction. The bloom formation mechanism requires a comprehensive consideration of multiple factors, including concentrations of nutrients such as nitrogen and phosphorus, water temperature, light conditions, water flow, and the ecological carrying capacity of the river [5]. Research on cyanobacterial blooms in urban rivers has been extensively studied and exhibits distinct characteristics compared to lake ecosystems. Urban rivers, especially river mouths, where nutrients accumulate, also face risks of bloom outbreaks [6]. The formation of cyanobacterial blooms results from multiple interacting factors, with excessive nutrient inputs (nitrogen, phosphorus, etc.) being the most critical, while light and hydrological conditions are also important for harmful algae proliferation [7]. Under global warming, changes in climate and precipitation patterns intensify fluctuations in nutrient concentrations, exacerbating the complexity and frequency of cyanobacterial blooms and seriously threatening ecosystem stability and sustainability [8]. The driving mechanisms of urban river cyanobacterial blooms exhibit significant spatial heterogeneity [9]. At river mouths, longer water retention times, salinity gradients, and sediment resuspension cause lake-like stratification, promoting vertical migration and aggregation of toxin-producing algae such as Microcystis [10]. In addition to river mouths, land use type is another important factor influencing bloom outbreaks. Long-term analyses by international scholars have found a positive correlation between bloom intensity and agricultural land area [11]. Both river mouths and agricultural areas are nutrient-enriched segments. Researchers have attempted to predict bloom risk using nutrient thresholds; especially in highly urbanized rivers, fluctuations in nutrient levels correlate significantly with cyanobacterial dominance [12,13]. Moreover, bloom risks vary seasonally; high temperatures and strong sunlight in summer favor rapid algal growth, and summer–autumn rains introduce excess nutrients into surface runoff, further increasing bloom risks [14]. Urban rivers also show pulsed pollution inputs during rainy seasons, potentially disrupting algal community niches and triggering short-term blooms.

Cyanobacterial blooms are highly complex aquatic phenomena, and their formation mechanisms and physiological traits are also major research focuses. Bloom formation involves adaptive regulation of phytoplankton under environmental stress. Physiological studies mainly concentrate on nutrient utilization, photosynthetic efficiency, antioxidant capacity, and toxin metabolism [15,16]. Different algae species exhibit significant differences in nutrient uptake and resource use efficiency, directly affecting bloom occurrence [17]. Photosynthesis research reveals that algae adjust their photosynthetic systems to maintain efficient light use under ecological competition and changing hydrological conditions [18]. Recently, research on algal toxin metabolism and ecological effects—especially microcystin production, secretion patterns, and interactions with environmental factors—has provided important theoretical bases for bloom risk assessment [19,20]. These studies deepen understanding of bloom physiology and support monitoring, prediction, and control strategies.

Current bloom monitoring methods mainly fall into traditional and modern categories. Traditional methods include water sampling and laboratory analysis relying on microscopy, chlorophyll-a measurement, and physicochemical indicators (e.g., nitrogen, phosphorus concentrations). Although accurate, these methods are time consuming, labor intensive, and limited in spatial coverage [21]. Modern approaches such as remote sensing and drone monitoring combined with ecological models enable large-scale, real-time bloom monitoring [22]. Multispectral and hyperspectral remote sensing can rapidly identify spatiotemporal algal distributions, especially effective in turbid waters [23]. Management practices generally assess bloom severity by cyanobacterial cell density and implement source control, ecological restoration, and emergency response accordingly [24]. Source control focuses on reducing nutrient inputs by improving agricultural fertilization techniques and constructing wetlands to decrease non-point source pollution [25]. Ecological restoration enhances water self-purification through food web regulation and aquatic vegetation recovery. Emergency responses, during bloom outbreaks, use aeration and chemical algaecides to rapidly reduce algal biomass and prevent further deterioration [26]. Integrated multidisciplinary bloom management is becoming mainstream, but challenges remain, including model uncertainties, high economic costs, and complex ecosystem recovery post-treatment [27]. Future efforts should integrate multisource monitoring data, optimize prediction models, and combine ecological engineering with social management for efficient, sustainable bloom control [28].

Among bloom mechanism studies, remote sensing is the most popular macro-level method but lacks small-scale analysis capacity [29,30]. Molecular biological monitoring offers a novel early warning approach, opening new perspectives [31]. In addition to remote sensing and molecular methods, various ecological models such as partial least squares structural equation modeling, Bayesian methods, and generalized additive models have been used to study blooms [32]. Phytoplankton resource use efficiency is an emerging indicator to decipher urban river cyanobacterial bloom mechanisms and correlates with cyanobacterial dominance [33,34]. Research shows that excessive cyanobacterial growth increases toxin levels, severely damages aquatic community structures and ecosystem functions, and significantly alters phytoplankton resource use efficiency [35]. Cyanobacterial dominance is regulated by multiple factors, closely related to physiological traits and significantly associated with nutrient concentrations, temperature, and nitrogen-to-phosphorus ratio [36,37]. With urbanization and excessive nutrient discharge, increasing evidence indicates rising bloom risks, significantly impacting aquatic community structure and resource use efficiency [38,39]. Human disturbances alter relationships between phytoplankton diversity and ecosystem function, complicating links between nutrient levels and phytoplankton resource use efficiency [40]. Aquatic community structure and phytoplankton resource use efficiency exhibit nonlinear relationships influenced by other factors like water temperature. Thus, this study employed generalized additive models to analyze bloom mechanisms from the perspective of resource use efficiency across river segments [41].

## 2. Materials and Methods

### 2.1. Study Area

The Majiagou River (126°41′–126°43′ E, 45°32′–45°49′ N) is a first-order tributary located on the southern bank of the Songhua River. It originates in Wanghuiwen Village, Lixin Township, Acheng District, Harbin, and flows for a total length of 44.3 km, with 34.7 km running through the urban area. The upstream Gongnong Reservoir is a small-sized reservoir with a storage capacity of 3.1 million m^3^. It primarily functions as a water supply source for the urban river channel, while also serving roles in flood control, irrigation, fisheries, and tourism. The Majiagou River flows through key urban districts of Harbin, including Pingfang, Xiangfang, Nangang, Daoli, and Daowai, making it one of the most important inland rivers in this northeastern Chinese city. It represents a typical urban river system. Along its course from the source to the confluence with the Songhua River, the river traverses areas with diverse land use types, including rural, peri-urban, and urban landscapes. This diversity in topography and land use across the watershed contributes to significant environmental heterogeneity along different river sections.

The region where the Majiagou River is located is characterized by a temperate continental monsoon climate, with long, cold winters and short, warm summers. The mean annual temperature in Harbin is approximately 5.6 °C, with the average temperature in January reaching −22 °C and extreme highs approaching 38 °C in summer. Due to the cold climate, the Majiagou River typically freezes completely from late November to February each year, lasting 2 to 3 months, exhibiting typical features of rivers in cold regions. Harbin receives an average annual precipitation of 569.1 mm, with more than 60% falling during the summer months (June to August). As a result, the Majiagou River displays pronounced seasonal variation in runoff and hydrological processes, with high flows during the summer flood season and flow interruption due to freezing during winter.

### 2.2. Sample Collection

Based on the climatic characteristics and hydrological conditions of the Majiagou River basin, this study conducted eight sampling campaigns of aquatic organisms and water quality during the following periods: May 2022 (spring), July 2022 (summer), September 2022 (autumn), November 2022 (Ice Formation Period), March 2023 (Thawing Period), May 2024 (spring), July 2024 (summer), and September 2024 (autumn).

**Figure 1:**
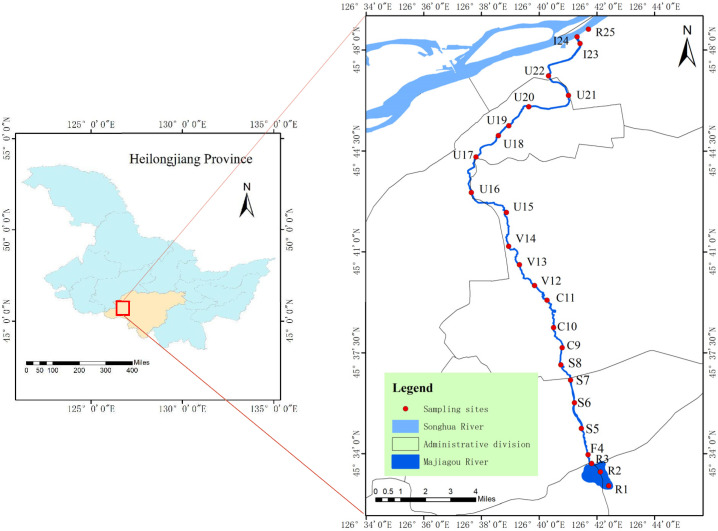
Study area and sampling point locations.

According to the river’s actual hydrological and ecological conditions and following the principles of representativeness, controllability, and uniformity based on water body scale, 25 sampling sites were selected (Figure 1). The sampling locations remained consistent across all sampling periods. At each site, samples of phytoplankton, zooplankton, benthic macroinvertebrates, and water environmental parameters were collected. Due to the shallow depth of the river, no stratified sampling was conducted. Given that most sections of the Majiagou River freeze completely in winter and are too shallow for fish to overwinter, fish surveys were only carried out in the upstream reservoir and river mouth areas. Specifically, the sampling points were distributed across the following zones: reservoir and fishery areas (R1–R3, F4), agricultural areas (S5–S8), rural and small industrial zones (C9–C11), vegetation restoration zones (V12–V14), urban areas (U15–U22), river mouth (I23–I24), and the Songhua River (R25). The reservoir and river mouth areas (R1–R3, I23–I24) are dominated by muddy substrates; the upper and middle reaches (S5–S8, C9–C11, V12–V14) are primarily gravel-bottomed or rich in humus sediment; fishery and urban sections (F4, U15–U22) are characterized by channelized banks; and the Songhua River section (R25) has a sandy-gravel bottom. Each river section is subject to different levels of human disturbance and pressure, leading to substantial spatial and seasonal variations in aquatic organisms and environmental parameters.

### 2.3. Data Analysis

During the field investigation, on-site measurements of Chl-a were conducted using a multi-parameter water quality analyzer (YSI 6600, YSI, Youngstown, OH, USA) to enable real-time monitoring. The determination of TN and TP was executed using specific methodologies, including the alkaline potassium persulfate digestion–UV spectrophotometric method for TN and ammonium molybdate spectrophotometry for TP. For phytoplankton, Water samples were collected using a 1L plexiglass sampler at a depth of 0.5 m, and 1.5% Lugol’s solution was added for fixation. Samples were left to settle for 48 h, after which the supernatant was siphoned off, and the remaining sample volume was adjusted to 50 mL in polyethylene bottles. Algal identification and counting were conducted using a 10 × 40 optical microscope, with diatom samples undergoing acid treatment before examination under a 10 × 100 microscope. Phytoplankton dominance (*Y*) was assessed using the McNaughton dominance index.

(1) $$Y=N_{i}/N\times f_{i}$$

*Y* represents the species dominance index; *Nᵢ* is the density of the target species (cells/L); *N* is the total density of the community (cells/L); and *fᵢ* is the occurrence frequency of the species (*fᵢ* = number of samples in which the species occurred/total number of samples).

In our study, relative abundance was defined as the proportion of cell density of each phytoplankton taxon or functional group relative to the total cell density within a sample. Relative abundance was chosen for further analysis to emphasize community compositional shifts and functional group dynamics across sites and seasons, rather than absolute bloom magnitude. This approach allowed us to identify environmental drivers of ecological dominance while minimizing the confounding effects of fluctuations in total biomass. RUE was used to reflect the conversion efficiency of nutrients (TP and TN) by algae, in order to analyze the relationship between nutrient availability and algal community structure from the perspective of nutrient utilization. The RUEs of TN and TP were calculated as the ratio of chlorophyll-a concentration to TN and TP concentrations, respectively. The calculation formula is as follows:

(2) $$RUE=\frac{Chl-a(\mu g/L)}{TN(\mathrm{mg}/L)\cdot or\cdot TP(\mathrm{mg}/L)}$$

where Chl-a represents the chlorophyll-a concentration (μg/L), and TN and TP are the concentrations of TN and TP in the water (mg/L), respectively. RUE can be used to compare nutrient utilization efficiency across sites or seasons, with higher values indicating more effective conversion of available nutrients into algal biomass. It also helps identify potential nutrient limitations by revealing whether increased nutrient availability leads to proportional increases in chlorophyll-a.

The Generalized Additive Model (GAM) is a non-parametric regression approach extended from the Generalized Linear Model (GLM), which introduces nonlinear smoothing functions to capture complex nonlinear relationships between variables. This modeling technique is well-suited for analyzing data with non-normal distributions or scenarios where nonlinear patterns exist between predictors and response variables. In this study, the GAM was employed to elucidate the response relationships between cyanobacterial relative abundance and environmental nutrients, as well as phytoplankton resource use efficiency. In the model settings, the default gamma value was used, and continuous predictor variables were assigned a maximum of four degrees of freedom, with a minimum smoothing parameter set to 3. To compare model performance and select the optimal GAM structure, the Akaike Information Criterion (AIC) and Generalized Cross Validation (GCV) were applied. Several key environmental variables and their interaction terms were included in the models. The final selected model ensured that residuals satisfied or approximated a normal distribution, thereby improving model validity and interpretability [42,43]. The GAM can be used to flexibly model nonlinear relationships between predictors and response variables, allowing better capture of ecological patterns than traditional linear models. By applying smoothing functions and model selection criteria such as AIC and GCV, the GAM helps identify key drivers while ensuring model robustness and interpretability. The application of the GAM in this study was based on preliminary exploratory analyses, which indicated potential nonlinear relationships between cyanobacterial relative abundance, nutrient concentrations, and resource use efficiency. Given the seasonal variability and complex interactions among environmental variables in the river ecosystem, the GAM provided the flexibility to model these nonlinear patterns without imposing strict parametric assumptions, making it particularly suitable for capturing ecological processes driving bloom dynamics across river sections.

The general structure of a GAM is expressed as

(3) $$g(E(Y))=\beta_{0}+f_{1}(x_{1})+f_{2}(x_{2})+\cdots +f_{m}(x_{m})$$

where *g*(*g*(.)) is the link function, *E*(*Y*) represents the expected value of the response variable, *β*_0_ is the intercept, and *f_i_*(*x_i_*) are the smoothing functions of the predictors. All GAM analyses in this study were performed using the mgcv package in R.

## 3. Results

### 3.1. Algal Communities and Water Quality

**Figure 2:**
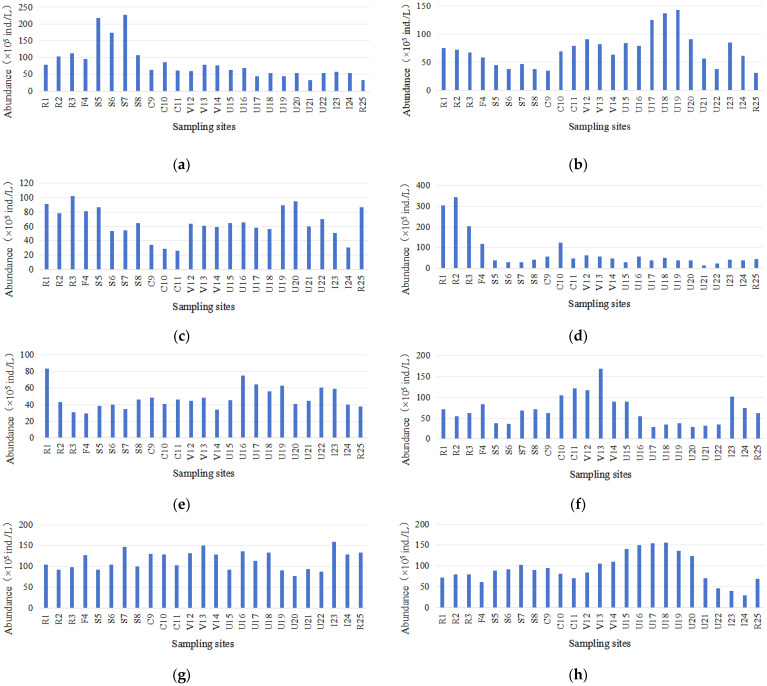
Temporal and spatial distributions of phytoplankton abundance in the Majiagou River. (**a**) May 2022, (**b**) May 2024, (**c**) July 2022, (**d**) July 2024, (**e**) September 2022, (**f**) September 2024, (**g**) Ice Formation Period, (**h**) Thawing Period.

Based on the results of eight surveys, a total of 134 species of phytoplankton belonging to 70 genera and eight phyla were identified in the Majiagou River (Figure 2). Among them, Chlorophyta had the highest species richness, with 52 species across 27 genera, accounting for 38.81% of the total. This was followed by Bacillariophyta, with 41 species in 20 genera (30.60%), and Cyanophyta, with 18 species in 11 genera (13.43%). Additionally, Euglenophyta included 11 species in 4 genera (8.21%); Dinophyta, 4 species in 3 genera (2.99%); Cryptophyta and Chrysophyta each had 3 species in 2 genera (2.24%); and Xanthophyta had 2 species in 1 genus (1.49%). Phytoplankton abundance ranged from 2.84 × 10^6^ to 2.27 × 10^7^ ind./L in spring, 3.17 × 10^6^ to 1.58 × 10^7^ ind./L in summer, 2.57 × 10^6^ to 1.56 × 10^7^ ind./L in autumn, 1.29 × 10^6^ to 3.45 × 10^7^ ind./L during the ice-covered period, and 2.97 × 10^6^ to 8.35 × 10^6^ ind./L during the thawing period. Biomass values varied between 0.28 and 13.42 mg/L (spring), 0.32 and 4.2 mg/L (summer), 0.27 and 6.14 mg/L (autumn), 0.16 and 3.64 mg/L (ice-covered), and 0.2 and 3.96 mg/L (thawing).

A total of 13 common phytoplankton species were identified in the Majiagou River (Table 1). Among them, Bacillariophyta (diatoms) were the most abundant, with six species, including *Cyclotella meneghiniana*, *Synedra acusvar*, *Synedra ulna*, *Fragilaria intermedia*, *Stauroneis anceps*, and *Navicula radiosa*. This was followed by Cyanophyta (cyanobacteria), with four species, including *Raphidiopsis sinensis*, *Oscillatoria tenuis*, *Phormidium tenue*, and *Microcystis wesenbergii*. Chlorophyta (green algae) accounted for three species, including *Chlorella vulgaris*, *Platymonas subcordiformis*, and *Scenedesmus bicaudatus*. In terms of seasonal distribution, the number of common phytoplankton species was highest in spring and summer (eight species each), followed by autumn (six species), ice formation period (five species), and the lowest in the thawing period (three species). A total of 18 dominant phytoplankton species were identified in the Majiagou River, among which Bacillariophyta (diatoms) had the highest representation with 7 species, followed by Cyanophyta (cyanobacteria) with 6 species, and Chlorophyta (green algae) with 5 species. In spring, nine dominant species were recorded, with dominance indices ranging from 0.026 to 0.287. These included *Raphidiopsis sinensis*, *Chlorella vulgaris*, *Cyclotella meneghiniana*, *Synedra acus*, *Synedra ulna*, *Fragilaria intermedia*, *Stauroneis anceps*, *Spirulina platensis*, and *Platymonas subcordiformis*. The most dominant species was *Synedra ulna* (Spring 2022). In summer, eight dominant species were identified, with dominance indices ranging from 0.023 to 0.248, including *Oscillatoria tenuis*, *Scenedesmus bicaudatus*, *Chlorella vulgaris*, *Cyclotella meneghiniana*, *Synedra ulna*, *Synedra acus*, *Melosira varians*, and *Stauroneis anceps*. The highest dominance was observed for *Cyclotella meneghiniana* (Summer 2022). In autumn, 12 dominant species were found, with dominance indices ranging from 0.020 to 0.150. These included *Raphidiopsis sinensis*, *Platymonas subcordiformis*, *Scenedesmus bicaudatus*, *Navicula radiosa*, *Crucigenia tetrapedia*, *Chlorella vulgaris*, *Spirogyra communis*, *Cyclotella meneghiniana*, *Synedra acus*, *Synedra ulna*, *Melosira varians*, and *Ulothrix tenerrima*. The most dominant species was *Synedra acus* (Autumn 2024). During the ice formation period, five dominant species were observed, with dominance indices ranging from 0.024 to 0.254. These included *Phormidium tenue*, *Raphidiopsis sinensis*, *Cyclotella meneghiniana*, *Synedra ulna*, and *Stauroneis anceps*, with *Cyclotella meneghiniana* being the most dominant. In the thawing period, seven dominant species were identified, with dominance indices ranging from 0.022 to 0.120. These included *Phormidium tenue*, *Chroococcus tenax*, *Raphidiopsis sinensis*, *Microcystis wesenbergii*, *Chlorella vulgaris*, *Cyclotella meneghiniana*, and *Synedra acusvar*, with *Cyclotella meneghiniana* showing the highest dominance.

### 3.2. River Section Division Based on Hierarchical Clustering

**Figure 3:**
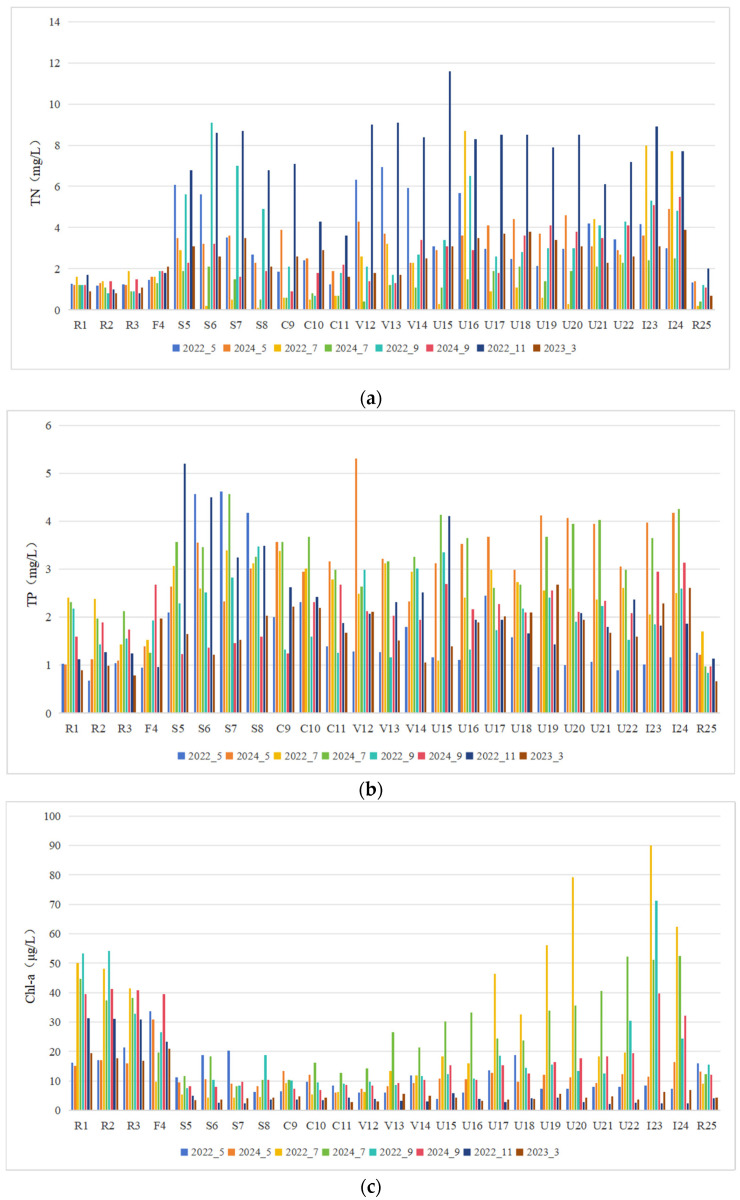
Spatial characteristics of nutrients and Chl-a at sampling sites in the Majiagou River. (**a**) TN, (**b**) TP, (**c**) Chl-a.

The analysis revealed significant spatial variations in TN, TP, and Chl-a concentrations across all sampling sites (*p* < 0.01, Figure 3). TN concentrations ranged from 0.1 to 11.6 mg/L (mean: 3.11 mg/L), with the maximum (11.6 mg/L) recorded at site U15 in November 2022 and the minimum (0.1 mg/L) at site S8 in July 2022. Similarly, TP concentrations varied between 0.66 and 5.31 mg/L (mean: 2.31 mg/L), peaking at site V12 in May 2024 (5.31 mg/L) and reaching the lowest level at site R25 in March 2023 (0.66 mg/L). Chlorophyll-a (Chl-a) concentrations exhibited the widest range (2.03–90.08 μg/L, mean: 16.33 μg/L), with the highest value (90.08 μg/L) observed at site I23 in July 2022 and the lowest (2.03 μg/L) at site U21 in November 2022. 

**Figure 4:**
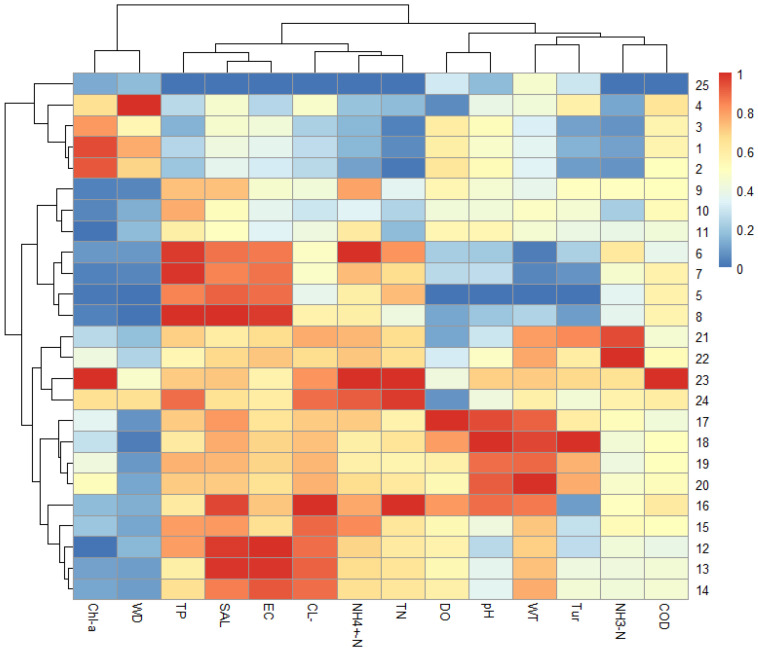
Division of river sections in Majiagou River based on hierarchical cluster analysis.

In this study, physicochemical indicators, including nutrient concentrations, were standardized, and Hierarchical Cluster Analysis (HCA) was applied to assess the similarity of water quality parameters across 25 sampling sites. Based on the clustering results (Figure 4), the Majiagou River was classified into three types of water bodies: (1) Reservoirs, fishery areas, and the Songhua River: Sampling sites R1–R3, F4, and R25; (2) Rural and suburban areas: Sampling sites S5–S8 and C9–C11; (3) Urban areas: Sampling sites V12–V14, U15–U22, and I23–I24. The classification derived from the hierarchical cluster analysis generally aligns with the actual hydrological conditions. As this study primarily focuses on urban river ecosystems, food web modeling was not conducted for the sites located in the reservoir, fishery area, and the Songhua River. Therefore, the Majiagou River was further divided into rural/suburban and urban river sections. Notably, in the urban section, especially near the river mouth, nutrients tend to accumulate, and dissolved oxygen concentrations are relatively low.

### 3.3. Spatiotemporal Variations in the Relative Abundance of Cyanobacteria

Abundance is an effective indicator of phytoplankton community structure. In this study, the relative abundance of cyanobacteria was used to reflect their dominance at each monitoring site. The dominant cyanobacterial genera in the Majiagou River include Microcystis, Chroococcus, Anabaena, Oscillatoria, Anabaenopsis, and Aphanizomenon. Cyanobacteria exhibited clear spatiotemporal variations; the relative abundance of cyanobacteria was higher in the urban reaches than in the rural and suburban reaches. The highest relative abundance occurred at site I24 in September 2022 (autumn), reaching 40.65%. In spring, cyanobacterial relative abundance was generally low in both river sections, ranging from 0.29% to 25.71%, with an average of 8.56%. The highest value was observed at site I24 in May 2024 (25.71%), and the lowest at site S7 (0.39%). In summer, cyanobacterial relative abundance increased compared to spring, ranging from 3.62% to 30.63%, with an average of 14.19%. The highest value occurred at site I23 in July 2024 (30.63%), and the lowest at site C10 in July 2022 (3.62%). In autumn, the relative abundance of cyanobacteria was highest across both river sections, ranging from 3.29% to 40.65%, with an average of 16.03%. The peak was at site I23 in September 2022 (40.65%), while the lowest value was at site U15 in September 2024 (3.29%). During the Ice Formation Period, the overall relative abundance was slightly higher than in spring, ranging from 3.73% to 17.65%, with an average of 10.55%. The highest values were recorded at sites S6 (17.65%) and S5 (15.59%), while the lowest was at site C10 (3.73%). During the Thawing Period, the overall relative abundance of cyanobacteria was comparable to that in summer, ranging from 4.24% to 20.30%, with an average of 14.02%. The highest value was at site I23 (20.30%), followed by site V12 (18.11%), and the lowest was at site S5 (4.24%).

### 3.4. Spatial and Temporal Variations in Resource Use Efficiency

This study used RUE to reflect the conversion efficiency of algae for TP and TN, analyzing their correlation with algal community structure from the perspective of nutrient elements. The resource use efficiency of TN and TP was calculated as the ratio of chlorophyll-a concentration to TN and TP concentrations, respectively. In addition to the resource use efficiency of TN and TP, the study also considered the spatial and temporal distribution differences of the nitrogen-to-phosphorus ratio (N/P). Independent sample *t*-tests were conducted to analyze the differences in resource use efficiency and N/P between the two river segments, as shown in Table 2. The resource use efficiency of TN did not exhibit significant differences between the two river segments (*p* > 0.05), whereas significant differences were found in the resource use efficiency of TP and N/P (*p* < 0.05). Overall, the resource use efficiency of TN and TP in urban river segments was lower than that in rural and suburban river segments, while the N/P ratio showed the opposite trend.

**Figure 5:**
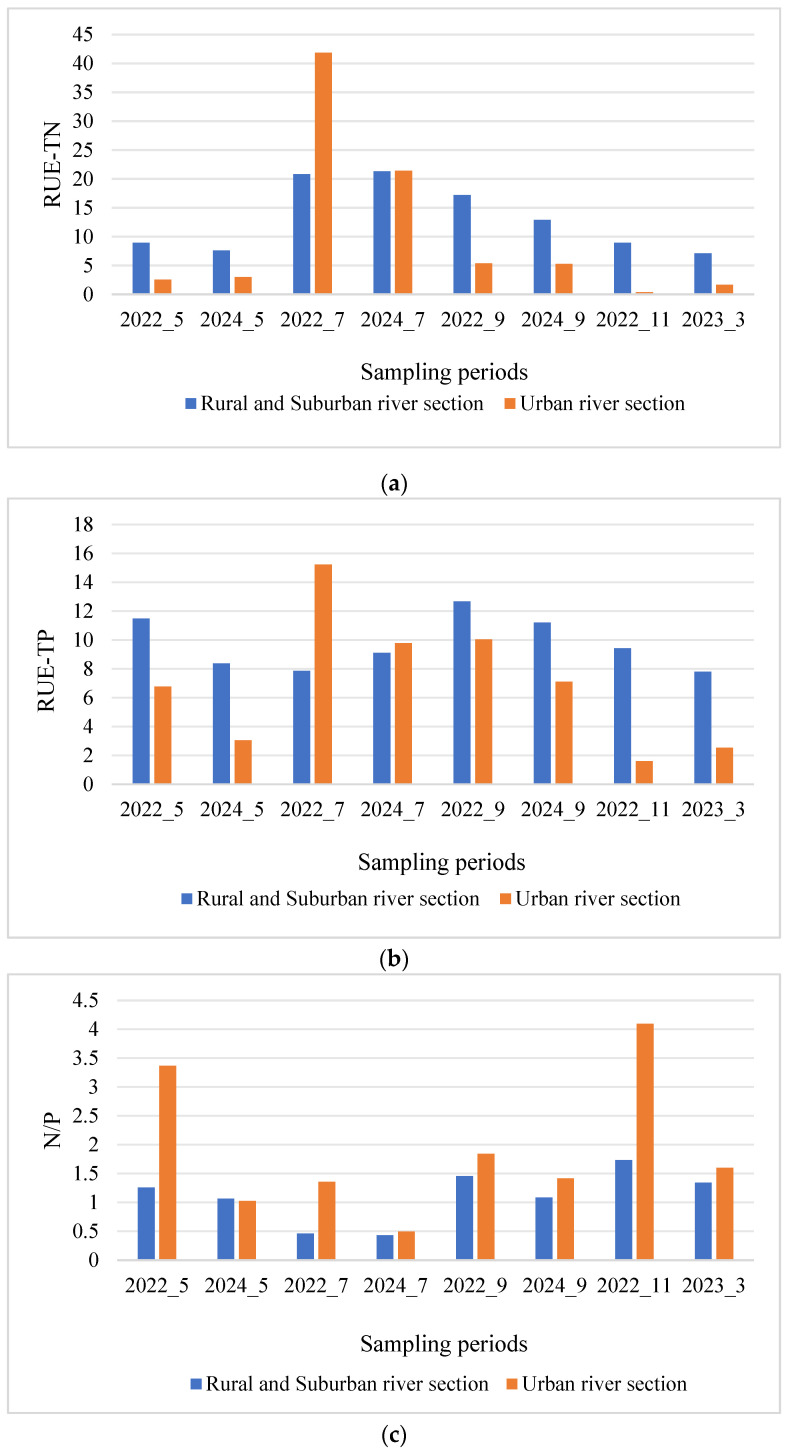
TN and TP resource utilization efficiency and N/P in two types of river sections of the Majiagou River during different periods: (**a**) RUE-TN, (**b**) RUE-TP, (**c**) N/P.

This study also used one-way ANOVA to analyze the differences in resource use efficiency and N/P across different sampling periods. First, homogeneity of variance and Shapiro–Wilk normality tests were conducted to determine the most appropriate one-way ANOVA method. The results of the homogeneity test showed that the variance of TN resource use efficiency and N/P failed to meet the homogeneity requirement, so a more robust Welch’s ANOVA method was applied. As shown in Table 3 and Figure 5, both resource use efficiency and N/P exhibited significant spatial differences (*p* < 0.05), with post hoc multiple comparisons performed using the LSD method. Overall, the resource use efficiency of TN in summer was significantly higher than in other periods, with the highest mean value of 41.85 occurring in July 2022 in the urban river segment. The second-highest mean value also occurred in summer. The resource use efficiency of TN was lower during the ice formation and thawing periods, with the lowest mean value of 0.388 occurring in the urban river segment during the ice formation period. Similarly, TP resource use efficiency was higher in summer and autumn, with the highest mean value of 15.24 occurring in July 2022 and the lowest in the ice formation and thawing periods, with the lowest value of 1.6 occurring in the urban river segment during the ice formation period. The N/P ratio was highest during the ice formation period, with the highest mean value of 4.09 occurring in the urban river segment during this period, followed by the spring urban river segment with a mean of 3.37. The N/P ratio was lowest in summer, with the lowest mean value of 0.43 occurring in July 2024.

### 3.5. Analysis of Cyanobacterial Impact Mechanisms Based on Resource Use Efficiency

#### 3.5.1. Construction and Selection of GAMs for Rural and Suburban River Sections

**Figure 6:**
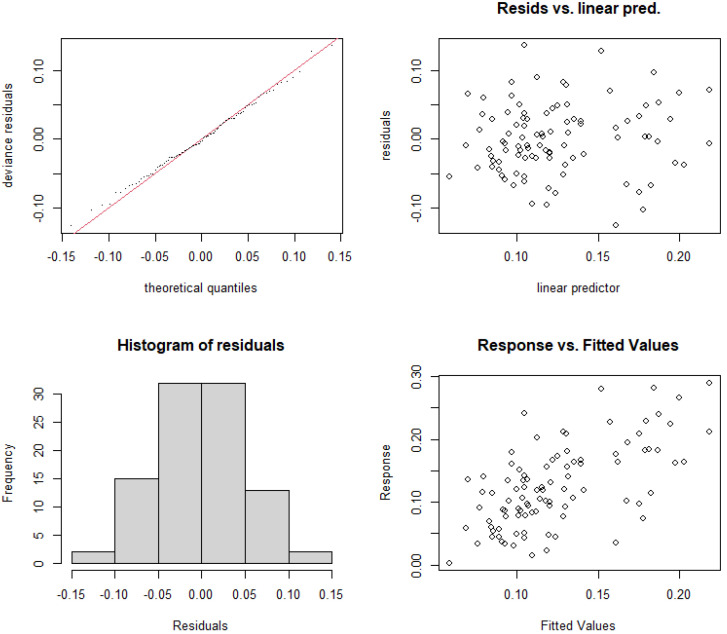
Residual analysis of the nonlinear model structure for relative abundance of cyanobacteria and RUE_TN with interaction terms added (Model 6).

**Figure 7:**
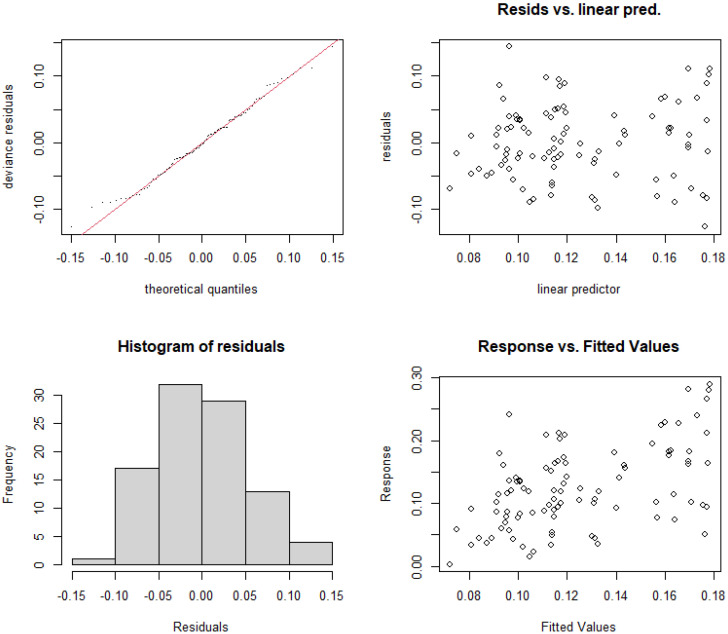
Residual analysis of the nonlinear model structure for relative abundance of cyanobacteria and RUE_TP with interaction terms added (Model 7).

Based on nutrient concentrations and resource use efficiency, this study constructed nonlinear models for the relative abundance of cyanobacteria in rural and suburban river sections of the Majiagou River. First, the significance of each individual model was tested, as shown in Table 4, and the results indicate that the RUE_TN, RUE_TP, and N/P indicators passed the significance test (*p* < 0.05). Next, considering the complex coupling relationships between explanatory variables, interaction terms were introduced to improve the model’s robustness. After incorporating interaction terms, the variance explanation of the models increased to 39.5% and 36.4%. Additionally, the Akaike Information Criterion (AIC) and Generalized Cross-Validation (GCV) were used to assess the model’s robustness. The results show that with the addition of interaction terms, both the AIC and GCV values decreased, indicating an improvement in model accuracy. Finally, the residual histograms (Figure 6 and Figure 7) were tested to evaluate the model’s robustness, and the results show that the residuals of Model 6 and Model 7, after adding the interaction terms, approximately follow a normal distribution.

#### 3.5.2. Nonlinear Analysis of Cyanobacteria Relative Abundance and Resource Use Efficiency in Rural and Suburban River Sections

**Figure 8:**
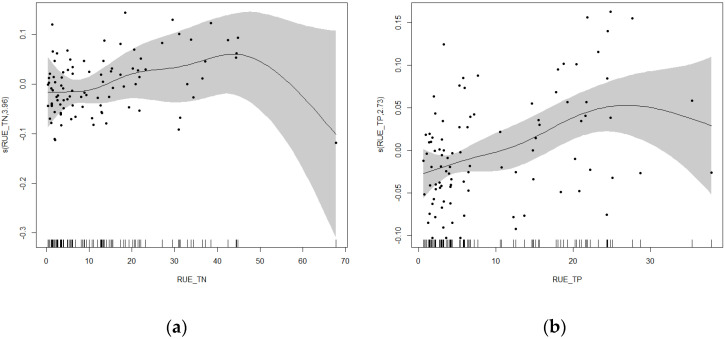
Response curve of cyanobacteria relative abundance to TN and TP resource utilization efficiency in rural and suburban river sections. (**a**) TN resource utilization efficiency, (**b**) TP resource utilization efficiency.

The results of the GAM confirmed the hypothesis of this study: the resource use efficiency of nutrients (TN, TP) shows a significant nonlinear relationship with the relative abundance of cyanobacteria (Figure 8). In the rural and suburban river sections of the Majiagou River, the response relationships between cyanobacteria relative abundance and the resource use efficiency of TN and TP exhibited both similarities and significant differences. Overall, RUE for TN and TP exhibited peaks in cyanobacterial relative abundance within specific ranges, below which both showed positive correlations. When the RUE of TN ranged from 40 to 45, the relative abundance of cyanobacteria was higher. Below this range, there was a positive correlation; above this threshold, correlation becomes difficult to determine due to limited sample size. For the RUE of TP, the highest relative abundance of cyanobacteria occurred when it ranged from 25 to 30. Below this range, there was a positive correlation; above this threshold, similar to TN, correlation becomes difficult to determine due to limited sample size.

#### 3.5.3. Construction and Selection of GAMs for Urban River Sections

**Figure 9:**
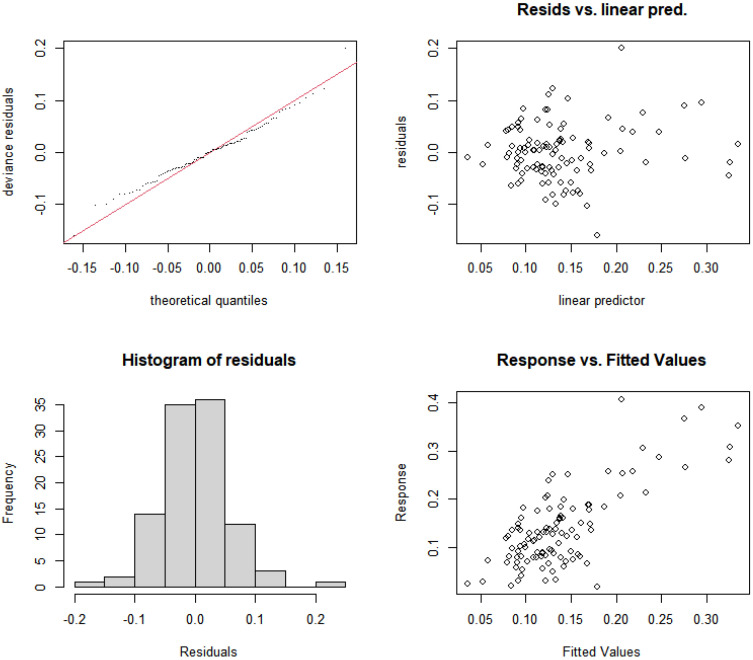
Residual analysis of the nonlinear model structure for relative abundance of cyanobacteria and RUE_TN with interaction terms added (Model 6).

**Figure 10:**
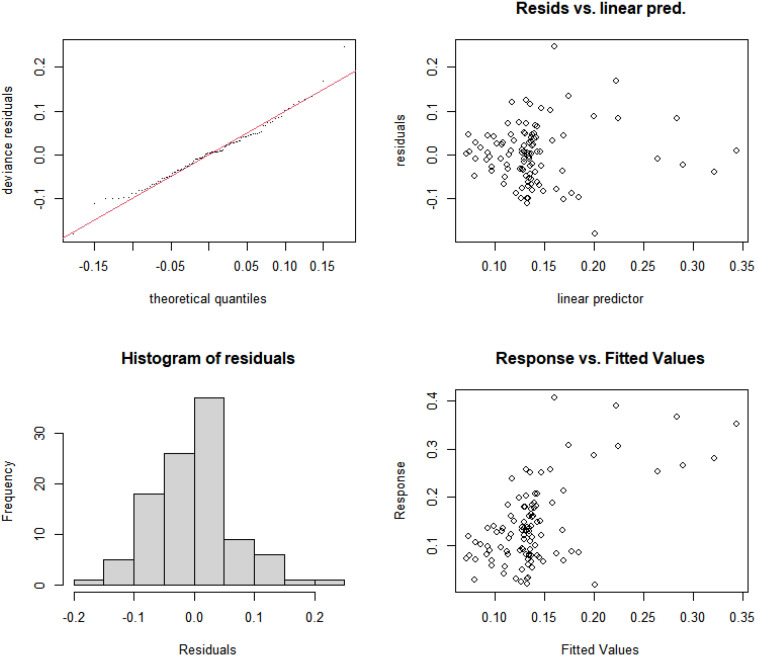
Residual analysis of the nonlinear model structure for relative abundance of cyanobacteria and RUE_TP with interaction terms added (Model 7).

Similar to the rural and suburban river sections, a nonlinear model for the relative abundance of cyanobacteria in the urban river section of the Majiagou River was constructed based on nutrient concentrations and their resource use efficiencies. As shown in Table 5, significance tests were conducted for each independent model, and the results indicated that TN concentration, TP concentration, RUE_TN, and RUE_TP passed the significance test (*p* < 0.05). As in the rural and suburban river sections, interaction terms for the explanatory variables were introduced, and the model was evaluated using AIC and GCV values. The results show that after introducing the interaction terms, the model’s variance explanation increased significantly, from 16.6% and 18.2% to 56% and 54.7%, respectively. Additionally, both AIC and GCV values decreased, indicating that the model’s accuracy improved. Based on the residual histograms (Figure 9 and Figure 10), the residuals of Model 6 and Model 7 with interaction terms were approximately normally distributed, allowing for further analysis.

#### 3.5.4. Nonlinear Analysis of Cyanobacteria Relative Abundance and Resource Use Efficiency in Urban River Sections

**Figure 11:**
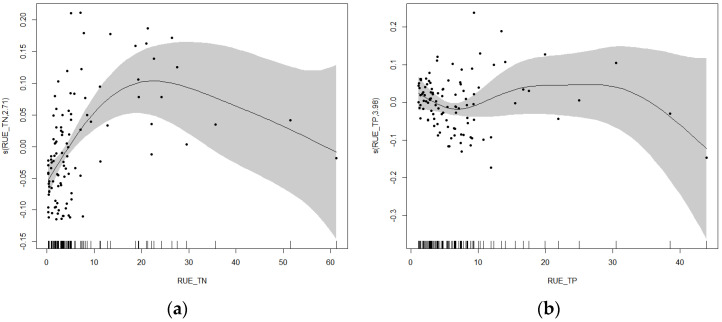
Response curve of cyanobacteria relative abundance to (**a**) TN and (**b**) TP resource utilization efficiency in urban river section.

Similar to the rural and suburban river sections, the resource use efficiencies of nutrients (TN, TP) in the urban river section show a significant nonlinear relationship with the relative abundance of cyanobacteria (Figure 11). In the urban river section of the Majiagou River, the response relationship between cyanobacterial relative abundance and the resource use efficiencies of TN and TP show significant differences. The relationship between cyanobacterial relative abundance and TN resource use efficiency first increased and then decreased. When the TN resource use efficiency ranged from 20 to 25, the relative abundance of cyanobacteria was higher; below this range, a positive correlation was observed, and above this range, a negative correlation was found. The relationship between cyanobacterial relative abundance and TP resource use efficiency was more complex. When the TP resource use efficiency was below approximately 10, a negative correlation was observed, and when it was above this value, the response showed a slight initial increase followed by a decline, with the increase being relatively stable. Compared to the rural and suburban river sections, the threshold for the response change between cyanobacterial relative abundance and TN resource use efficiency in the urban river section was notably lower. Below the threshold, the positive correlation was more pronounced in the urban river section, while it was weaker in the rural and suburban sections. The response relationship between cyanobacterial relative abundance and TP resource use efficiency in the urban river section showed significant differences compared to the rural and suburban sections. When TP resource use efficiency was low, the response relationships between the two river sections were opposite.

## 4. Discussion

Our study conducted an investigation and analysis of the spatiotemporal differences in cyanobacterial relative abundance, nutrient concentrations, and their resource use efficiencies across different sections of the Majiagou River. Generalized Additive Models (GAMs) were constructed to analyze the nonlinear relationships between cyanobacterial relative abundance and nutrient resource use efficiencies, revealing the optimal ranges for cyanobacterial bloom outbreaks. Phytoplankton resource use efficiency is an important indicator of how phytoplankton communities in aquatic systems respond to environmental changes, and its spatiotemporal variations are jointly influenced by factors such as nutrient concentrations, light intensity, water temperature, and biological pressures from zooplankton and benthic macroinvertebrates [44]. Phytoplankton resource use efficiency exhibits significant differences across temporal and spatial scales, reflecting the complexity of nutrient cycling and biotic interactions within aquatic ecosystems. In this study, we discuss the spatiotemporal variations in phytoplankton resource use efficiency and their driving factors from multiple perspectives.

### 4.1. The Driving Effects of Environmental Factors on Phytoplankton Resource Use Efficiency

Phytoplankton resource use efficiency reflects the capacity of primary producers to convert key nutrients such as TN and TP into biomass. Nutrient concentrations are among the decisive factors influencing phytoplankton growth, particularly TN and TP, as their levels directly affect photosynthetic rates and growth processes, thereby shaping resource use efficiency [45]. In this study, TN and TP concentrations exhibited pronounced seasonal fluctuations. It is inferred that abundant precipitation during summer and autumn facilitated nutrient input into the water bodies, leading to generally higher nutrient concentrations. During these periods, phytoplankton effectively absorbed and utilized these nutrients, resulting in elevated resource use efficiency. In contrast, during winter and spring, lower temperatures and limited light availability constrained photosynthesis, markedly reducing nutrient absorption and conversion efficiency and consequently diminishing resource use efficiency.

Division of river sections reflects differences in land use, pollution sources, and hydrological characteristics along the river. Rural/suburban sections are influenced mainly by agricultural runoff and small-scale industry, while urban sections are affected by concentrated domestic sewage and stormwater inputs. These distinctions provide a basis for analyzing spatial variations in nutrient dynamics and phytoplankton community structure. Spatial variations in nutrient concentrations across regions also influenced the spatial distribution of phytoplankton resource use efficiency [46]. In rural and suburban river sections, faster flow rates led to relatively lower nutrient concentrations, whereas in urban areas, slower water flow promoted nutrient accumulation. Moreover, water temperature and light intensity directly affect phytoplankton photosynthetic efficiency, which in turn determines their resource use efficiency [47]. Research has shown that both light intensity and temperature exhibit significant seasonal variability, influencing phytoplankton photosynthesis and growth [48]. In this study, higher temperatures and stronger light intensity during summer and autumn provided optimal growth conditions, resulting in higher resource use efficiency. Conversely, in spring, the ice formation period, and the thawing period, low temperatures and weak light intensity significantly suppressed photosynthesis, leading to reduced resource use efficiency.

Beyond seasonal changes, water transparency and depth also affect the light conditions available to phytoplankton [49]. For instance, in river sections with higher water transparency, phytoplankton received more light, thereby enhancing photosynthesis and increasing resource use efficiency. In contrast, in areas with low transparency, limited light penetration restricted resource use efficiency. Hydrological characteristics further influenced phytoplankton community structure and their resource use efficiency. Relatively stable flow conditions enabled phytoplankton to consistently absorb nutrients, whereas abrupt changes in flow could dilute or precipitate nutrients, impeding efficient utilization. Differences in flow stability between regions contributed to significant spatial variations in resource use efficiency between rural–suburban and urban river sections.

### 4.2. Mechanistic Analysis of Factors Driving Cyanobacterial Blooms

Cyanobacterial blooms are one of the typical manifestations of eutrophication in aquatic environments and commonly occur in waters with high nutrient concentrations. Cyanobacteria have broad requirements for nitrogen and phosphorus, making them prone to bloom outbreaks in nutrient-rich waters. Elevated water temperatures promote cyanobacterial growth, while anthropogenic activities such as agricultural discharge and urban runoff further exacerbate nutrient inputs, intensifying bloom formation. In this study, the relative abundance of cyanobacteria increased significantly during summer and autumn, closely associated with eutrophication phenomena. It is inferred that high TN and TP concentrations provided sufficient nutrient sources to support rapid cyanobacterial growth. Particularly in warmer seasons, rising temperatures and enhanced light availability further promoted cyanobacterial reproduction, resulting in increased relative abundance.

Beyond nutrient concentrations and temperature–light conditions, water flow velocity is another critical factor influencing bloom occurrence. In rural and suburban river sections, faster flow rates hindered cyanobacteria from forming blooms. Conversely, in urban river sections, especially near estuarine areas, slower flow rates coupled with severe eutrophication allowed cyanobacteria to accumulate and proliferate rapidly, leading to bloom events. Furthermore, the buoyancy regulation mechanisms unique to cyanobacteria enabled them to aggregate near the water surface, securing optimal light conditions and enhancing photosynthetic efficiency.

The GAM results revealed that cyanobacterial relative abundance exhibited distinct nonlinear responses to TN and TP resource use efficiencies. Within certain efficiency thresholds, increases in nutrient use efficiency corresponded to elevated cyanobacterial growth; however, beyond these thresholds, relative abundance gradually declined. This indicates that cyanobacterial dominance depends not only on the absolute concentrations of nutrients but also on the utilization capabilities of phytoplankton for these nutrients. In particular, in urban river sections, nutrient oversupply and eutrophication often coincide with reduced phytoplankton utilization efficiency, creating favorable conditions for rapid cyanobacterial proliferation and increasing the risk of bloom outbreaks.

### 4.3. Seasonal and Sectional Nonlinear Responses of Cyanobacterial Abundance to Resource Use Efficiency

Phytoplankton resource use efficiency is jointly influenced by community structure, interspecific competition, and environmental factors. Cyanobacteria compete for resources with other aquatic organisms. Due to their nitrogen-fixing ability, low photosynthetic saturation point, and efficient nutrient absorption mechanisms, cyanobacteria hold a competitive advantage in high-nutrient environments. When phytoplankton resource use efficiency is low, cyanobacteria can rapidly grow and reproduce, quickly utilizing available nutrients. This competitive relationship exhibits notable variations across temporal and spatial scales. In water bodies dominated by cyanobacteria, the overall resource use efficiency of phytoplankton tends to decrease, likely because cyanobacteria enhance their competitive capacity for critical resources such as light and nutrients, putting other phytoplankton groups like green algae and diatoms at a disadvantage [50]. During cyanobacterial blooms, the excessive growth of cyanobacteria often depletes large amounts of nutrients, leading to a decline in the overall biomass of phytoplankton, which significantly affects resource use efficiency. In bloom periods, phytoplankton communities dominated by cyanobacteria exhibit lower species diversity and functional balance.

Additionally, cyanobacteria’s ability to effectively absorb and store nutrients further strengthens their competitive advantage for nitrogen and phosphorus during bloom outbreaks, restricting the growth of other phytoplankton and reducing their resource use efficiency, thereby lowering the efficiency of energy and material transfer within the ecosystem [51]. For instance, under conditions of high nutrient concentrations and low phytoplankton resource use efficiency, cyanobacteria can proliferate rapidly, intensifying competition for phytoplankton resources and further reducing overall resource use efficiency in the water body. Moreover, cyanobacterial metabolites such as algal toxins also exert inhibitory effects on the growth of other phytoplankton, further diminishing overall resource use efficiency. Consequently, cyanobacterial blooms not only alter the energy flow patterns within aquatic ecosystems but also affect the stability and productivity of phytoplankton communities.

### 4.4. Ecological Management and Future Perspectives

Based on the analysis of phytoplankton RUE and its relationship with cyanobacterial relative abundance in this study, several implications can be drawn for ecological restoration and water quality management in urban rivers. (1) Nutrient management: Given the close association between cyanobacterial blooms and TP and TN inputs, it is recommended to strengthen the interception of agricultural non-point source pollution and enhance nitrogen and phosphorus removal in urban wastewater treatment to reduce excessive nutrient inputs and optimize the nutrient structure of water bodies. (2) Ecological restoration strategies: In urban river sections, improving hydrological conditions (e.g., increasing flow velocity and reducing suspended solids concentrations) and restoring riparian vegetation can enhance the self-purification capacity of water bodies, thereby promoting phytoplankton resource use efficiency and fundamentally regulating cyanobacterial dominance. (3) Dynamic monitoring and risk warning: It is suggested to establish high-frequency monitoring systems to track changes in phytoplankton RUE and cyanobacterial relative abundance in real time. By employing nonlinear models to predict bloom outbreak risks, timely emergency measures can be implemented to prevent cyanobacterial blooms. Future research should further integrate dynamic monitoring data, refine model parameters, and consider additional interaction factors (such as light intensity and emerging pollutants like microplastics) to comprehensively elucidate the driving mechanisms of cyanobacterial blooms, thereby providing a solid theoretical and technical foundation for the precise management of urban river ecosystems.

## 5. Conclusions

The results of this study indicate that the seasonal fluctuations in TN and TP concentrations directly influenced the nutrient absorption and conversion efficiency of phytoplankton, as reflected by changes in RUE. During summer and autumn, elevated temperatures and abundant sunlight significantly enhanced RUE, thereby providing favorable conditions for the rapid proliferation of cyanobacteria. Comparing rural/suburban and urban river sections, RUE was generally lower in urban areas due to slower water flow and greater nutrient deposition, while the relative abundance of cyanobacteria remained higher than in rural and suburban sections, suggesting that cyanobacteria possess a strong competitive advantage under eutrophic conditions and can maintain high dominance even when overall RUE is reduced.

## Figures and Tables

**Table 1:** Common phytoplankton species in Majiagou River.

Period	Phylum	Family	Species
Spring	Cyanophyta	Pseudanabaenaceae	*Raphidiopsis sinensis*
		Chlorellaceae	*Chlorella vulgaris*
	Bacillariophyta	Coscinodiscaceae	*Cyclotella meneghiniana*
		Fragilariaceae	*Synedra acusvar*
			*Synedra ulna*
			*Fragilaria intermedia*
		Stauroneidaceae	*Stauroneis anceps*
	Chlorophyta	Polyblepharidaceae	*Platymonas subcordiformis*
Summer	Cyanophyta	Oscillatoriaceae	*Oscillatoria tenuis*
		Phormidiaceae	*Phormidium tenue*
	Chlorophyta	Scenedesmaceae	*Scenedesmus bicaudatus*
		Chlorellaceae	*Chlorella vulgaris*
	Bacillariophyta	Coscinodiscaceae	*Cyclotella meneghiniana*
		Fragilariaceae	*Synedra ulna*
			*Synedra acusvar*
		Stauroneidaceae	*Stauroneis anceps*
Autumn	Cyanophyta	Pseudanabaenaceae	*Raphidiopsis sinensis*
	Chlorophyta	Scenedesmaceae	*Scenedesmus bicaudatus*
	Bacillariophyta	Naviculaceae	*Navicula radiosa*
		Coscinodiscaceae	*Cyclotella meneghiniana*
		Fragilariaceae	*Synedra acusvar*
			*Synedra ulna*
Ice Formation Period	Cyanophyta	Phormidiaceae	*Phormidium tenue*
		Pseudanabaenaceae	*Raphidiopsis sinensis*
	Bacillariophyta	Coscinodiscaceae	*Cyclotella meneghiniana*
		Fragilariaceae	*Synedra acusvar*
			*Synedra ulna*
Thawing Period	Cyanophyta	Pseudanabaenaceae	*Raphidiopsis sinensis*
		Microcystaceae	*Microcystis wesenbergii*
	Bacillariophyta	Fragilariaceae	*Synedra acusvar*

**Table 2:** Independent samples *t*-test: Analysis of differences between river sections.

	River Sections		Significance Test
	Rural and Suburban River Section	Urban River Section	*p*-Value
RUE-TN	13.11 ± 1.37	10.19 ± 2.78	0.685
RUE-TP	9.74 ± 0.93	7.02 ± 0.69	<0.05
N/P	1.11 ± 0.07	1.9 ± 0.14	<0.05

**Table 3:** One-way ANOVA: Analysis of differences among sampling periods.

	Seasons					Significance Test	
	Spring	Summer	Autumn	Freezing Period	Thawing Period	Statistics	*p*-Value
RUE-TN	5.41 ± 0.733	26.56 ± 5.42	9.99 ± 1.79	4.49 ± 2.02	4.29 ± 1.23	F = 9.142	<0.05
RUE-TP	7.33 ± 0.967	10.58 ± 1.26	10.19 ± 1.18	5.36 ± 1.79	5.07 ± 1.22	F = 3.48	<0.05
N/P	1.7 ± 0.18	0.7 ± 0.12	1.46 ± 0.12	2.96 ± 0.28	1.48 ± 0.09	F = 21.957	<0.05

**Table 4:** Independent models of explanatory variables and GAMs with added interaction terms.

GAMs	*p*-Value	GCV	Variance Explanation	AIC	Significance
Model1 RAC~s (TN, k = 3)	0.0736	0.00412	8.56%	−252.85	×
Model2 RAC~s (TP, k = 3)	0.0815	0.00415	8.51%	−252.16	×
Model3 RAC~s (RUE_TN, k = 3)	*p* < 0.001	0.00373	25.4%	−270.47	√
Model4 RAC~s (RUE_TP, k = 3)	*p* < 0.001	0.00364	20.3%	−264.93	√
Model5 RAC~s (N/P, k = 3)	*p* < 0.001	0.00436	0.532%	−247.31	√
Model6 RAC~s(RUE_TN) + te(TP,TN) + te(NP,RUE_TP)		0.00355	39.5%	−269.60	
Model7 RAC~s(RUE_TP) + te(TP,TN)		0.00354	36.4%	−263.66	

RAC: Relative abundance of cyanobacteria; √ Statistically significant; × Not significant.

**Table 5:** Independent models of explanatory variables and GAMs with added interaction terms.

GAMs	*p*-Value	GCV	Variance Explanation	AIC	Significance
Model 1: RAC~s (TN, k = 3)	*p* < 0.01	0.00603	25.5%	−235.26	√
Model 2: RAC~s (TP, k = 3)	*p* < 0.05	0.00656	5.95%	−225.70	√
Model 3: RAC~s (RUE_TN, k = 3)	*p* < 0.01	0.00607	16.6%	−233.85	√
Model 4: RAC~s (RUE_TP, k = 3)	*p* < 0.001	0.00571	18.2%	−240.16	√
Model 5: RAC~s (N/P, k = 3)	0.149	0.00673	4.99%	−223.03	×
Model 6: RAC~s(RUE_TN) + te(TP,TN)		0.00536	56%	−247.78	
Model 7: RAC~s(RUE_TP) + te(TP,TN)		0.00514	54.7%	−251.72	

RAC: Relative abundance of cyanobacteria; √ Statistically significant; × Not significant.

## Data Availability

The original contributions presented in the study are included in the article, further inquiries can be directed to the corresponding authors.
